# Supplementary figures and images for: Grassland restoration in typical wind-eroded regions effectively increase soil organic carbon
Source: PLoS One. 2026 Apr 9;21(4):e0346688. doi: 10.1371/journal.pone.0346688 (PMC13065026; doi:10.1371/journal.pone.0346688)

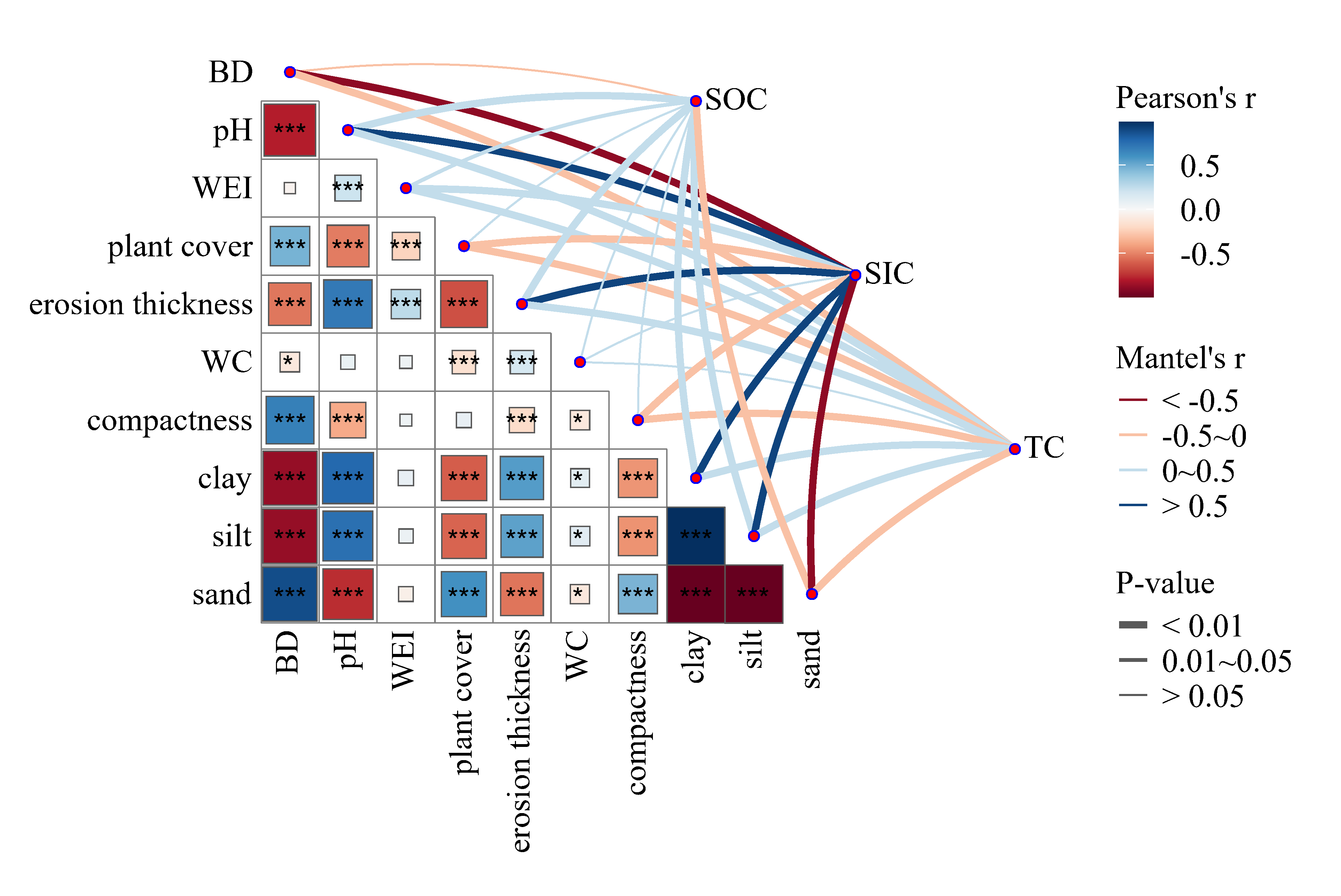

Supplement: S1 Fig — BD, bulk density (g cm-3); WEI, wind erosion index; WC, soil water content (%); SOC, soil organic carbon; SIC, soil inorganic carbon; TC, total carbon stock. The thickness of lines indicates the significance of Mantel tests, with thicker lines indicating more pronounced significance. The color of lines indicates the correlation coefficient of Mantel tests, while blue and red represent positive and negative correlations, respectively. *, **, *** means significant differences between layers at the 0.05, 0.01, 0.005 level, respectively. (TIF) [file pone.0346688.s001.tif]

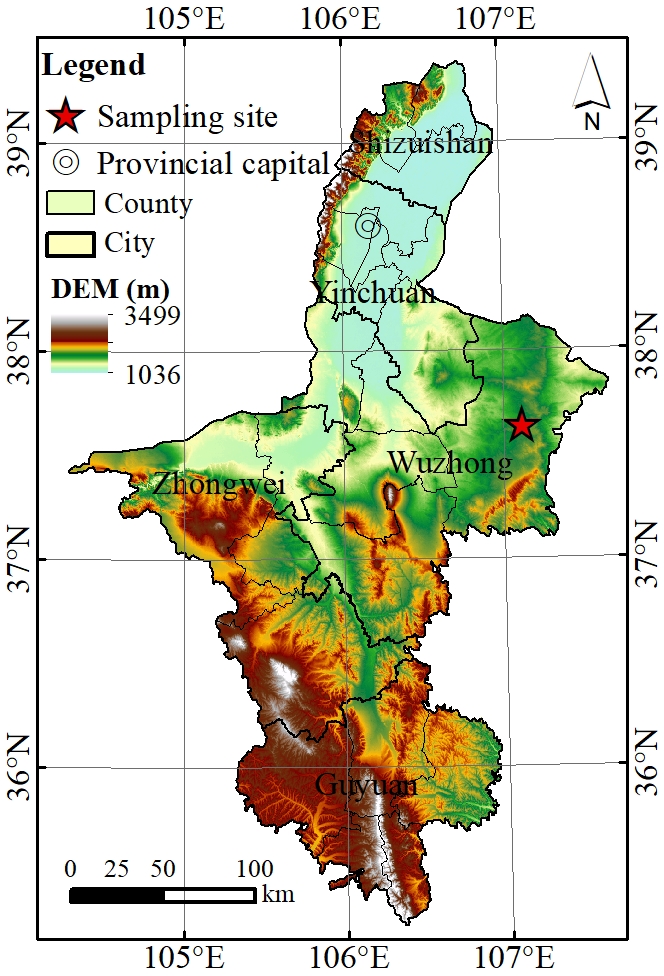

Supplement: S1 File — (ZIP) [file pone.0346688.s002.zip › Fig1.TIF]

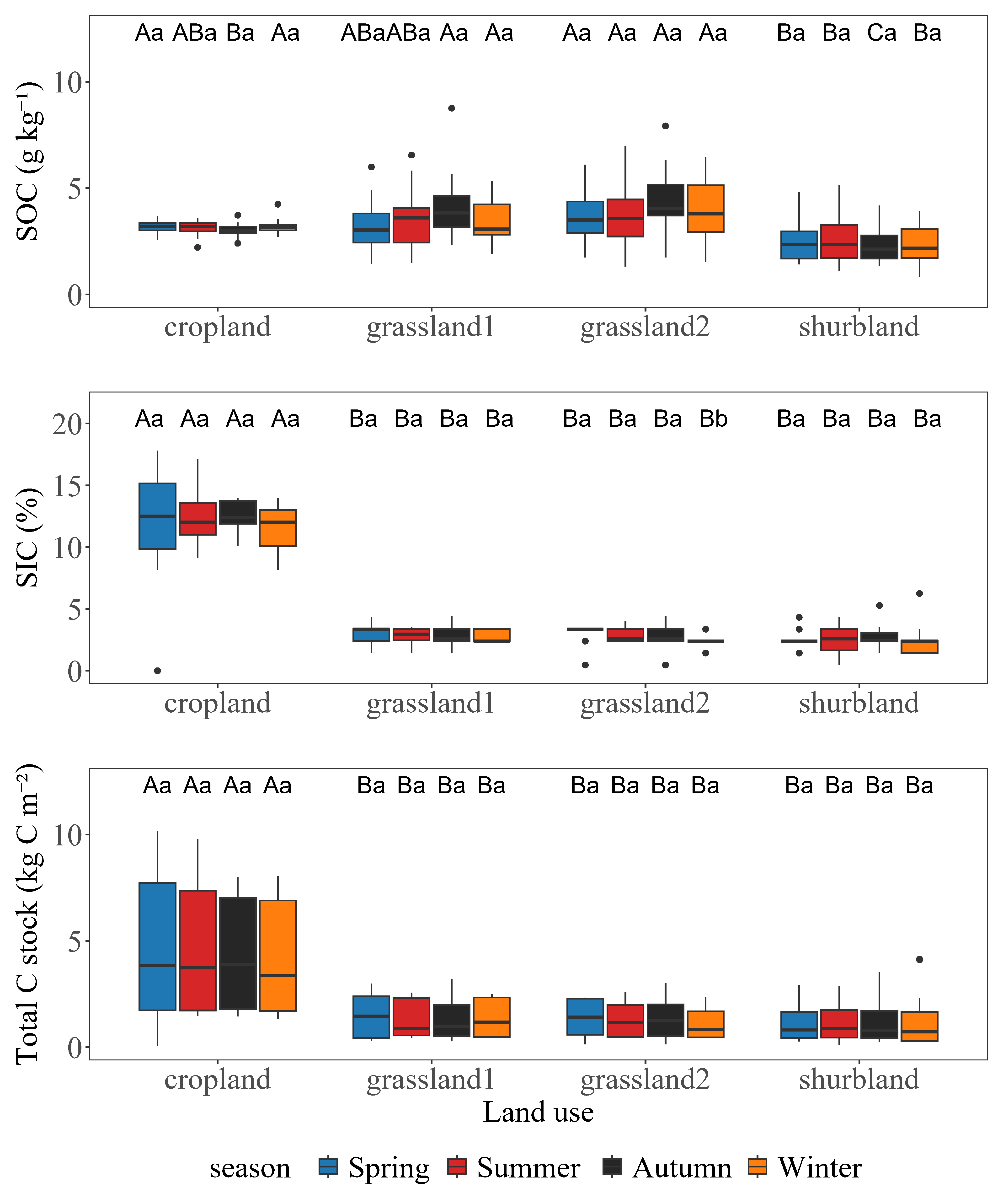

Supplement: S1 File — (ZIP) [file pone.0346688.s002.zip › Fig2.tif]

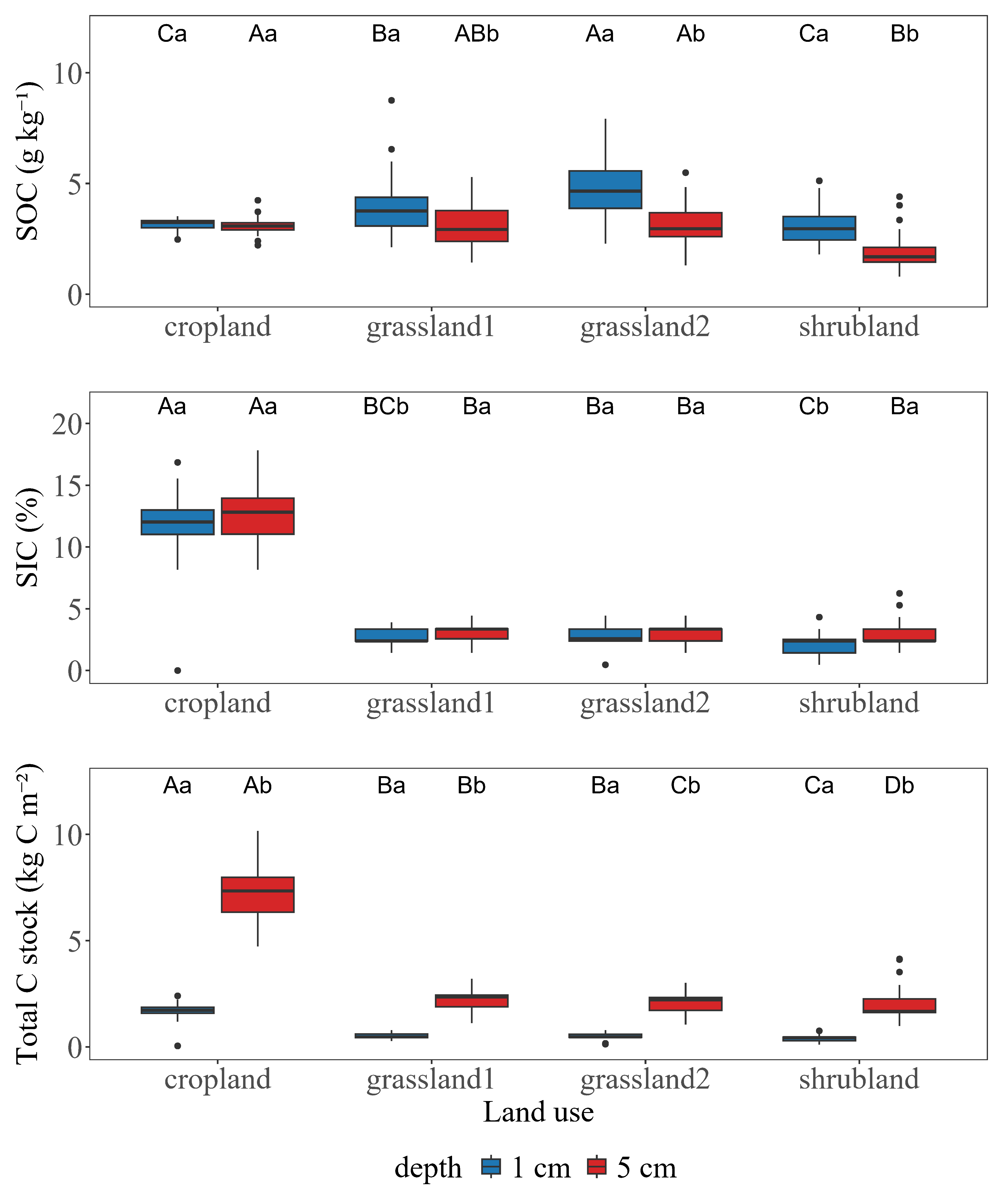

Supplement: S1 File — (ZIP) [file pone.0346688.s002.zip › Fig3.tif]

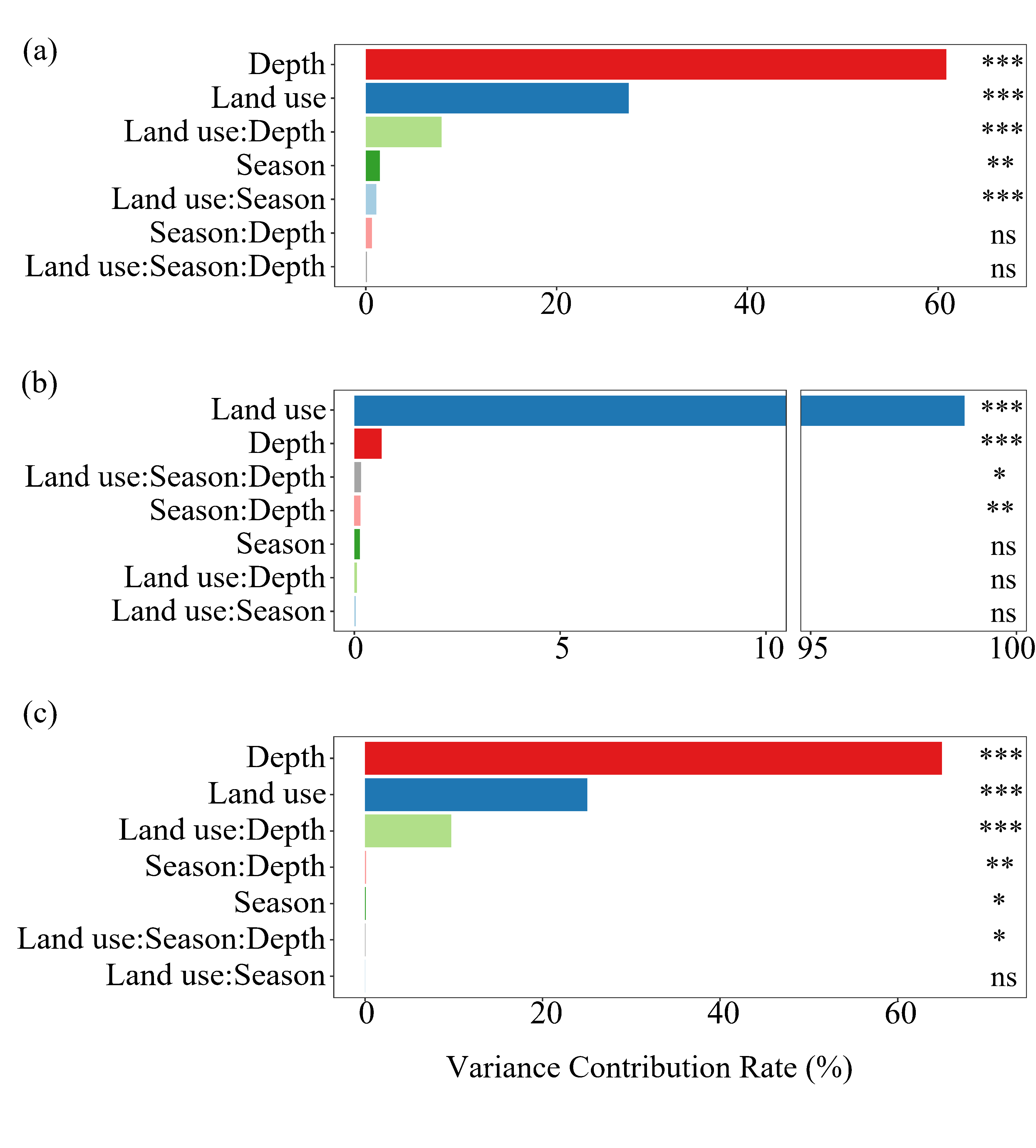

Supplement: S1 File — (ZIP) [file pone.0346688.s002.zip › Fig4.tif]

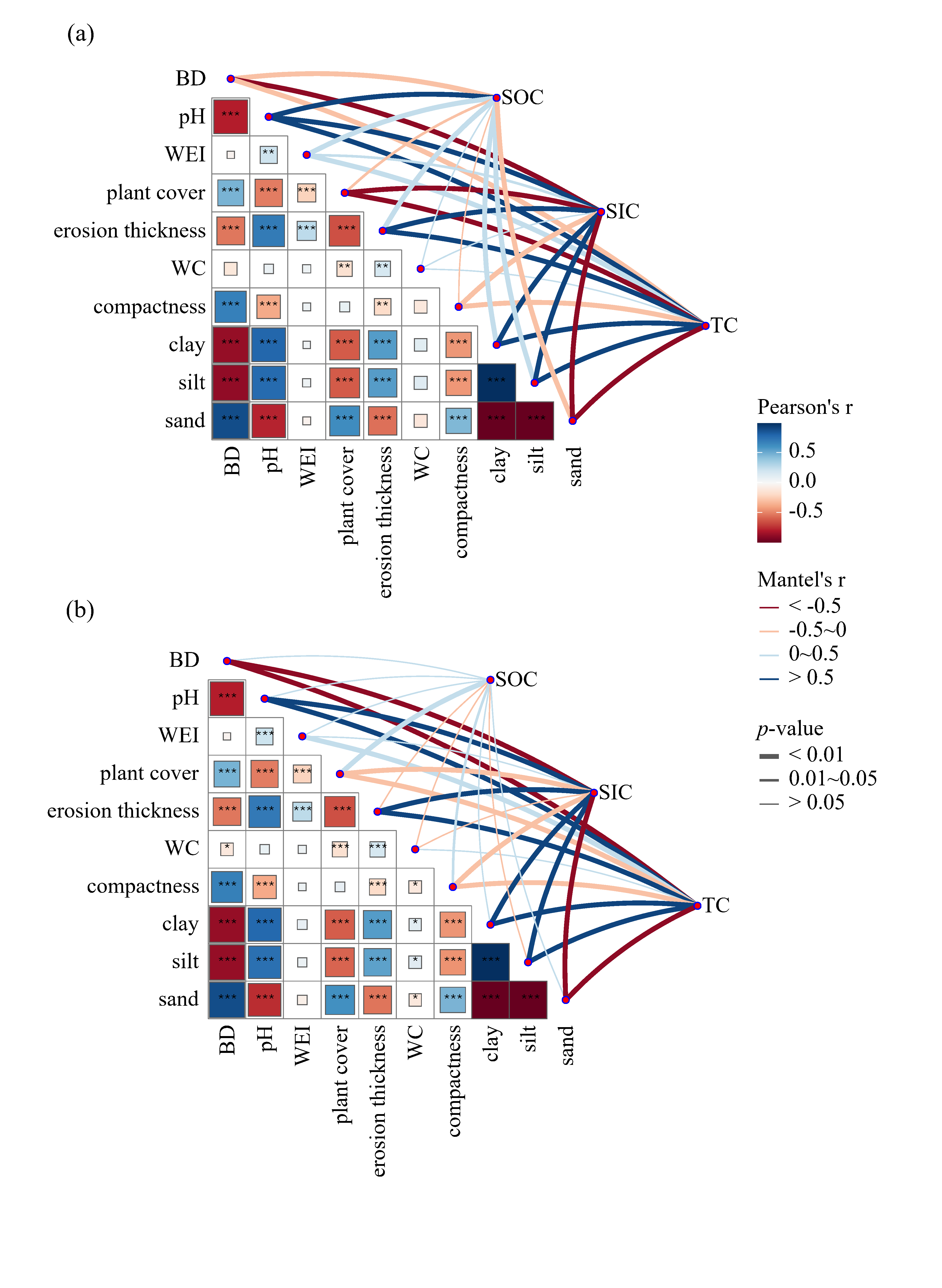

Supplement: S1 File — (ZIP) [file pone.0346688.s002.zip › Fig5.tif]

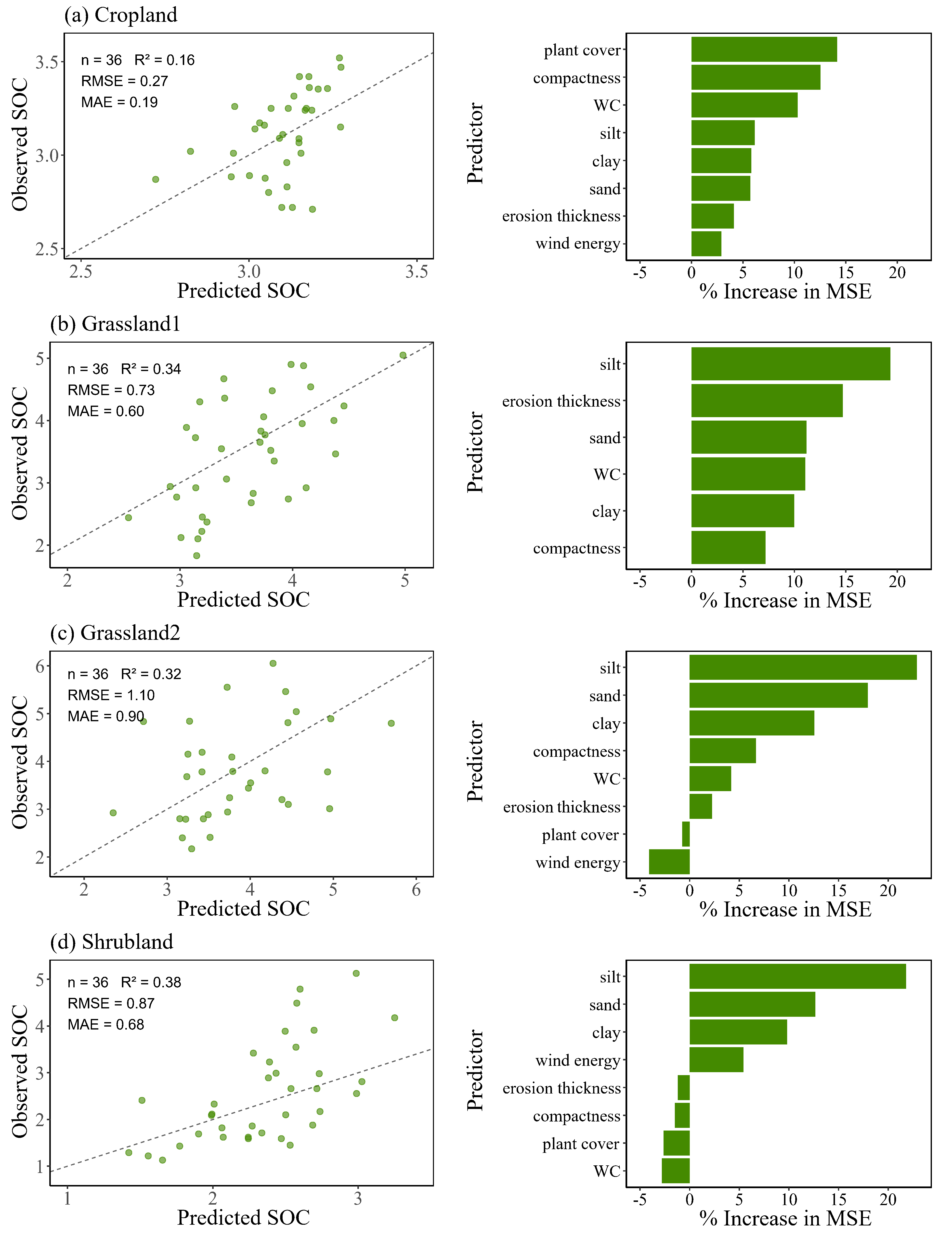

Supplement: S1 File — (ZIP) [file pone.0346688.s002.zip › Fig6.tif]

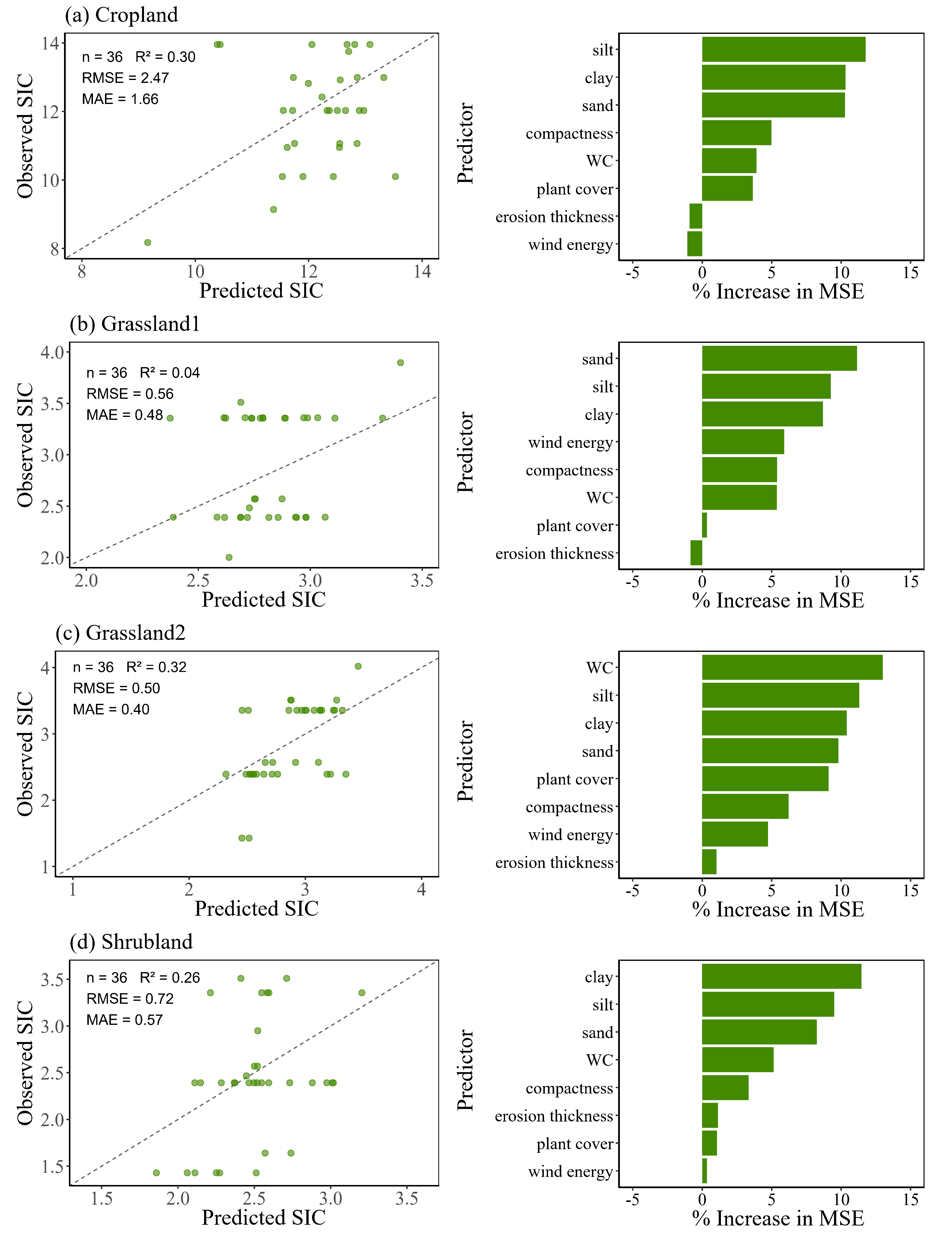

Supplement: S1 File — (ZIP) [file pone.0346688.s002.zip › Fig7.tif]

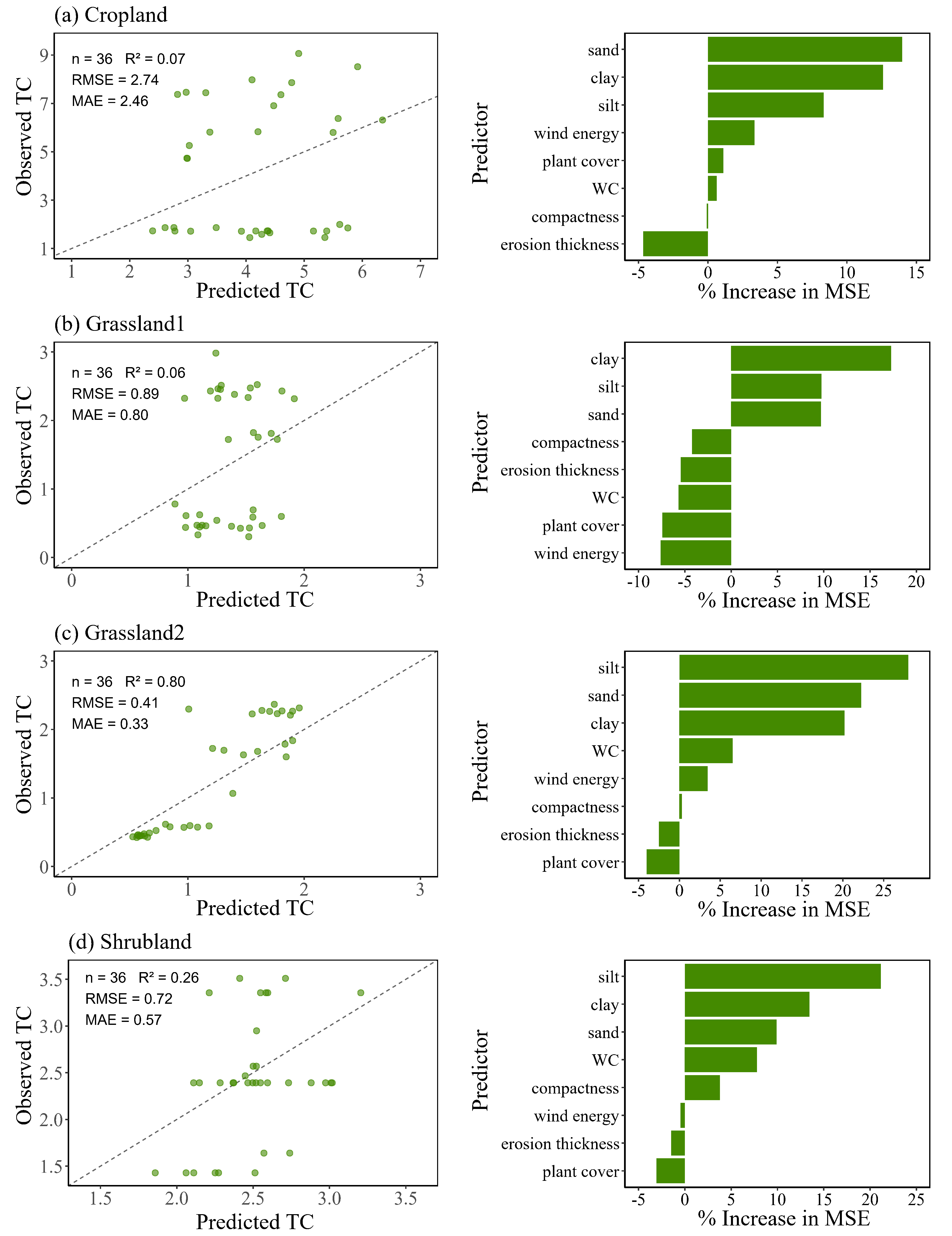

Supplement: S1 File — (ZIP) [file pone.0346688.s002.zip › Fig8.tif]
